# Supplementary material for: Multiscale Mathematical Modeling in Systems Biology: A Framework to Boost Plant Synthetic Biology
Source: Plants (Basel). 2025 Feb 5;14(3):470. doi: 10.3390/plants14030470 (PMC11820955; doi:10.3390/plants14030470)
Supplement: Supplementary file 1 [file plants-14-00470-s001.zip › plants-3298459-supplementary.pdf]

Table S1. List of databases for metabolic modeling in plants.

Database	Description	Data	Reference
BRENDA	BRENDA is a comprehensive relational database on functional and molecular information of enzymes	Enzyme (nomenclature and classification, function and properties, localization and expression, sources and organisms, kinetics and mechanisms)	[1]
KEGG	KEGG (Kyoto Encyclopedia of Genes and Genomes) is a knowledge base for systematic analysis of gene functions, linking genomic information with higher order functional information.	Pathway, functional orthologs, gene expressions, genomes, glycans, chemical compound, biochemical reactions	[2]
MetaCyc	MetaCyc is a curated database of experimentally elucidated metabolic pathways from all domains of life.	Metabolic pathways, chemical compounds and reactions, enzymes and genes	[3]
Metabolic Atlas	Metabolic Atlas is a web platform integrating open-source genome scale metabolic models (GEMs) for easy browsing and analysis	Metabolic pathways, enzyme reactions, genome, proteome, cellular localizations, regulatory networks	[4]
BioCyc	The BioCyc collection of Pathway/Genome Databases (PGDBs) provides a reference on the genomes, metabolic pathways, and (in some cases) regulatory networks of thousands of sequenced organisms.	Metabolic pathways, enzyme reactions, genome, gene function and regulation, comparative genomics	[5]
PMN	The PMN currently houses one multi-species reference database called PlantCyc and 126 species/taxon-specific databases.	Plant (metabolic pathway, genome)	[6]
UniProt	UniProt is the world's leading high-quality, comprehensive and freely accessible resource of	Protein(sequences, annotations, families and domains, localization and structure)	[7]

	protein sequence and functional information.			

Table S2. List of FSPM models categorized based on the Quantitative plant website [8]

Model name	Model features	Scale	Programming language	Reference
Shoot				
3dCAP	Shoot-architecture, resource acquisition, resource allocation, photosynthesis, light interception, environmental conditions	Shoot, whole plant, field		[9]
ALMANAC	Crop growth, yield, competition, light interception, biomass production, biomass allocation, water use, nutrient uptake, environmental conditions	Whole plant	Fortran	[10]
Agro-BGC	Photosynthesis, crop growth, crop development, fruits, yield, biomass production, management practices, phenology	Shoot, whole plant, field	C	[11]
AmapSim	Shoot architecture	Shoot, whole plant, field	C++	[12]
Arabidopsis	Shoot architecture, resource acquisition, resource allocation, photosynthesis	Shoot, whole plant, field	Java, RGG, XL	[13]
Arabidopsis Framework Model v2	Hypocotyl elongation, flowering time, rhythmic gene expression	Shoot, whole plant	Matlab	[14]
BarleyBreeeder	Shoot architecture, light interception, genetic	Shoot, whole plant, field	Java, RGG, XL	[15]

	recombination, mutation			
Brassica nigra	Shoot architecture, environmental conditions, resource acquisition, resource allocation, light interception, herbivory	Shoot, whole plant	Java	[16]
CottonXL	Shoot architecture, environmental conditions, resource acquisition, resource allocation, management practices, light interception	Shoot, whole plant, field	XL	[17]
Canopy Photosynthesi s	Shoot architecture, resource acquisition, photosynthesis, light interception, environmental conditions, management practices	Shoot, whole plant, field	Matlab, Java	[18]
EcoMeristem	Shoot architecture, resource allocation, carbohydrate metabolism, photosynthesis, light interception	Shoot, whole plant, field	C++, R	[19]
FSPM Soybean	Shoot architecture, photosynthesis, leaf transpiration, turgor pressure	Shoot, whole plant	Java	[20]
FSPM-P	Shoot architecture, environmental conditions, resource acquisition, resource allocation, management practices, light interception, photosynthesis	Shoot, whole plant, field	XL	[21]
GrapevineXL	Stomatal function, photosynthesis, water flux, berry-growth, environmental conditions	Shoot, whole plant	Java, R	[22]
GreenLab	Shoot architecture, light interception, photosynthesis	Shoot, whole plant, field	Matlab, Java, C++, Scilab	[23]

GOSSYM Cotton	Shoot architecture, environmental conditions, temperature, light interception, resource acquisition, resource allocation, photosynthesis, fruits	Shoot, whole plant, field		[24]
HydroShoot	Shoot architecture, gas exchange, canopy, water flux, carbon allocation, transpiration	Shoot, whole plant	Python	[25]
L-Cucumber	Shoot architecture, environmental conditions, resource acquisition, resource allocation, light interception, fruits	Shoot, whole plant, field	L, C	[26]
L-Donax	Shoot architecture, resource production	Shoot, whole plant	L, C	[27]
L-Tomato	Shoot architecture, light interception, shoot dry mass, environmental conditions	Whole plant, field	C++	[28]
MBPS-Apple	Photosynthesis	Shoot	Java	[29]
Phenomenal	Shoot architecture, light interception, radiation interception efficiency, radiation use efficiency	Shoot, whole plant, field	Python	[30]
Rice FSPM	Shoot architecture, photosynthesis, light interception	Shoot, whole plant, field	Java, RGG, XL	[31]
Rapeseed	Shoot architecture, resource acquisition, resource allocation, environmental conditions	Shoot, whole plant	Java, XL	[32]
V-Mango	Fruit production	Shoot, whole plant	C++, Python, R	[33]
SUNLAB	Biomass production, biomass allocation, environmental conditions	Shoot, whole plant, field	C++	[34]
Virtual Rose	Shoot architecture, resource acquisition, photosynthesis, light interception,	Shoot, whole plant, field	Java, XL	[35]

	management practices, environmental conditions			
Root				
ArchiSimple	RSA, shoot dry mass, environmental conditions	root	C, C++	[36]
CRootBox	RSA, environmental conditions, resource acquisition, resource allocation, water flux, containers	Root, whole plant, field	C++, Python	[37]
DigR	RSA, environmental conditions	Root, whole plant, field	C++, Java	[38]
OpenSimRoot	RSA, environmental conditions, resource acquisition, resource allocation, water flux, solute flux, shoot dry mass	Root, whole plant, field	C++	[39]
R-SWMS	RSA, water flux, solute flux, environmental conditions	Root, whole plant, field	C++, Fortran	[40]
ROOTMAP	RSA, environmental conditions, resource acquisition, resource allocation, water flux, containers	Root, whole plant, field	C++	[41]
Whole plant				
Alfalfa	Plant architecture, resource acquisition, resource allocation, photosynthesis	Root, shoot, whole plant, field		[42]
CN-Wheat	Plant architecture, resource acquisition, resource allocation, photosynthesis, environmental conditions, biomass allocation, biomass production	Root, shoot, whole plant	Python	[43]
CPlantBox	Plant architecture, water flux, solute flux, environmental conditions, carbon allocation	Root, shoot, whole plant	C++, Python, R	[44]
L-Almond	Plant architecture, shoot architecture,	Shoot, whole plant, field	L, C	[45]

	carbohydrate metabolism, resource acquisition, resource allocation			
L-egume	Plant architecture, resource acquisition, resource allocation, photosynthesis, water, nitrogen, plasticity, environmental conditions, management practices	Root, shoot, whole plant, field	Python	[46]
L-Peach	Shoot architecture, carbon production, resource allocation, management practices	Shoot, whole plant, field	L	[47]
LPJmL	Crop development, environmental conditions, management practices, photosynthesis, transpiration, resource allocation	Whole plant, field, global	C	[48]
MANIHOT-Cassava	Plant architecture, crop growth, temperature, biomass production, biomass allocation	Root, shoot, whole plant, field	Fortran	[49]
NEMA	Shoot architecture, resource acquisition, resource allocation, photosynthesis, nitrogen uptake, environmental conditions	Root, shoot, whole plant	C++	[50]
PiafMunch	Shoot architecture, solute flux, resource allocation, resource acquisition, vascular system, sinks-sources, transpiration	Root, shoot, whole plant	C++	[51]
PlaNet-Maize	Plant architecture, water flux, environmental conditions	Whole plant	Java, R	[52]
SPACSYS	RSA, resource acquisition, water flux, heat transfer	Root, shoot, field	C++	[53]

Table S3. Nutrient mobility in soil obtained from [54]

Element	Form	Soil mobility
Nitrogen (N)	NH_4^+	Immobile
	NO_3^-	Mobile
Phosphorus (P)	HPO_4^{2-}, $H_2PO_4^-$	Immobile
Potassium (K)	K^+	Low Mobility
Calcium (Ca)	Ca^{2+}	Low Mobility
Magnesium (Mg)	Mg^{2+}	Immobile
Sulfur (S)	SO_4^-	Mobile
Boron (B)	BO_3^-	Mobile
Copper (Cu)	Cu^{2+}	Immobile
Iron (Fe)	Fe^{2+}, Fe^{3+}	Immobile
Manganese (Mn)	Mn^{2+}	Mobile
Zinc (Zn)	Zn^{2+}	Immobile
Molybdenum (Mo)	MoO_4^-	Low Mobility
Chlorine (Cl)	Cl^-	Mobile
Cobalt (Co)	Co^{2+}	Low Mobility
Nickel (Ni)	Ni^{2+}	Low Mobility

References

1. Chang, A.; Jeske, L.; Ulbrich, S.; Hofmann, J.; Koblitz, J.; Schomburg, I.; Neumann-Schaal, M.; Jahn, D.; Schomburg, D. BRENDA, the ELIXIR Core Data Resource in 2021: New Developments and Updates. *Nucleic Acids Research* **2021**, *49*, D498–D508, doi:10.1093/nar/gkaa1025.
2. Kanehisa, M.; Furumichi, M.; Sato, Y.; Kawashima, M.; Ishiguro-Watanabe, M. KEGG for Taxonomy-Based Analysis of Pathways and Genomes. *Nucleic Acids Res* **2023**, *51*, D587–D592, doi:10.1093/nar/gkac963.
3. Caspi, R.; Altman, T.; Billington, R.; Dreher, K.; Foerster, H.; Fulcher, C.A.; Holland, T.A.; Keseler, I.M.; Kothari, A.; Kubo, A.; et al. The MetaCyc Database of Metabolic Pathways and Enzymes and the BioCyc Collection of Pathway/Genome Databases. *Nucleic Acids Research* **2014**, *42*, D459–D471, doi:10.1093/nar/gkt1103.
4. Li, F.; Chen, Y.; Anton, M.; Nielsen, J. GotEnzymes: An Extensive Database of Enzyme Parameter Predictions. *Nucleic Acids Research* **2023**, *51*, D583–D586, doi:10.1093/nar/gkac831.
5. Karp, P.D.; Billington, R.; Caspi, R.; Fulcher, C.A.; Latendresse, M.; Kothari, A.; Keseler, I.M.; Krummenacker, M.; Midford, P.E.; Ong, Q.; et al. The BioCyc Collection of Microbial Genomes and Metabolic Pathways. *Brief Bioinform* **2019**, *20*, 1085–1093, doi:10.1093/bib/bbx085.
6. Hawkins, C.; Ginzburg, D.; Zhao, K.; Dwyer, W.; Xue, B.; Xu, A.; Rice, S.; Cole, B.; Paley, S.; Karp, P.; et al. Plant Metabolic Network 15: A Resource of Genome-Wide Metabolism Databases for 126 Plants and Algae. *J Integr Plant Biol* **2021**, *63*, 1888–1905, doi:10.1111/jipb.13163.
7. The UniProt Consortium UniProt: The Universal Protein Knowledgebase in 2023. *Nucleic Acids Research* **2023**, *51*, D523–D531, doi:10.1093/nar/gkac1052.
8. Lobet, G.; Draye, X.; Périlleux, C. An Online Database for Plant Image Analysis Software Tools. *Plant Methods* **2013**, *9*, 38, doi:10.1186/1746-4811-9-38.
9. Chang, T.-G.; Zhao, H.; Wang, N.; Song, Q.-F.; Xiao, Y.; Qu, M.; Zhu, X.-G. A Three-Dimensional Canopy Photosynthesis Model in Rice with a Complete Description of the Canopy Architecture, Leaf Physiology, and Mechanical Properties. *Journal of Experimental Botany* **2019**, *70*, 2479–2490, doi:10.1093/jxb/ery430.

10. Kiniry, J.R.; Williams, J.R.; Gassman, P.W.; Debaeke, P. A General, Process-Oriented Model for Two Competing Plant Species. *Transactions of the ASAE* **1992**, *35*, 801–810, doi:10.13031/2013.28665.
11. Di Vittorio, A.V.; Anderson, R.S.; White, J.D.; Miller, N.L.; Running, S.W. Development and Optimization of an Agro-BGC Ecosystem Model for C4 Perennial Grasses. *Ecological Modelling* **2010**, *221*, 2038–2053, doi:10.1016/j.ecolmodel.2010.05.013.
12. Barczi, J.-F.; Rey, H.; Caraglio, Y.; de Reffye, P.; Barthélémy, D.; Dong, Q.X.; Fourcaud, T. AmapSim: A Structural Whole-Plant Simulator Based on Botanical Knowledge and Designed to Host External Functional Models. *Annals of Botany* **2008**, *101*, 1125–1138, doi:10.1093/aob/mcm194.
13. de Wit, M.; Kegge, W.; Evers, J.B.; Vergeer-van Eijk, M.H.; Gankema, P.; Voesenek, L.A.C.J.; Pierik, R. Plant Neighbor Detection through Touching Leaf Tips Precedes Phytochrome Signals. *Proceedings of the National Academy of Sciences* **2012**, *109*, 14705–14710, doi:10.1073/pnas.1205437109.
14. Chew, Y.H.; Seaton, D.D.; Mengin, V.; Flis, A.; Mugford, S.T.; George, G.M.; Moulin, M.; Hume, A.; Zeeman, S.C.; Fitzpatrick, T.B.; et al. The Arabidopsis Framework Model Version 2 Predicts the Organism-Level Effects of Circadian Clock Gene Mis-Regulation. *in silico Plants* **2022**, *4*, diac010, doi:10.1093/insilicoplants/diac010.
15. Buck-Sorlin, G.H.; Kniemeyer, O.; Kurth, W. Barley Morphology, Genetics and Hormonal Regulation of Internode Elongation Modelled by a Relational Growth Grammar. *New Phytologist* **2005**, *166*, 859–867, doi:10.1111/j.1469-8137.2005.01324.x.
16. de Vries, J.; Poelman, E.H.; Anten, N.; Evers, J.B. Elucidating the Interaction between Light Competition and Herbivore Feeding Patterns Using Functional–Structural Plant Modelling. *Annals of Botany* **2018**, *121*, 1019–1031, doi:10.1093/aob/mcx212.
17. Gu, S.; Evers, J.B.; Zhang, L.; Mao, L.; Zhang, S.; Zhao, X.; Liu, S.; van der Werf, W.; Li, Z. Modelling the Structural Response of Cotton Plants to Mepiquat Chloride and Population Density. *Annals of Botany* **2014**, *114*, 877–887, doi:10.1093/aob/mct309.
18. Song, Q.; Zhu, X.-G. A Model of Canopy Photosynthesis in Rice That Combines Sub-Models of 3D Plant Architecture, Radiation Transfer, Leaf Energy Balance and C3 Photosynthesis. In Proceedings of the 2012 IEEE 4th International Symposium on Plant Growth Modeling, Simulation, Visualization and Applications; October 2012; pp. 360–366.
19. Luquet, D.; Dingkuhn, M.; Kim, H.; Tambour, L.; Clement-Vidal, A. EcoMeristem, a Model of Morphogenesis and Competition among Sinks in Rice. 1. Concept, Validation and Sensitivity Analysis. *Functional Plant Biol.* **2006**, *33*, 309–323, doi:10.1071/FP05266.
20. Coussement, J.R.; De Swaef, T.; Lootens, P.; Steppe, K. Turgor-Driven Plant Growth Applied in a Soybean Functional–Structural Plant Model. *Annals of Botany* **2020**, *126*, 729–744, doi:10.1093/aob/mcaa076.
21. Henke, M.; Kurth, W.; Buck-Sorlin, G.H. FSPM-P: Towards a General Functional-Structural Plant Model for Robust and Comprehensive Model Development. *Front. Comput. Sci.* **2016**, *10*, 1103–1117, doi:10.1007/s11704-015-4472-8.
22. Zhu, J.; Dai, Z.; Vivin, P.; Gambetta, G.A.; Henke, M.; Peccoux, A.; Ollat, N.; Delrot, S. A 3-D Functional–Structural Grapevine Model That Couples the Dynamics of Water Transport with Leaf Gas Exchange. *Annals of Botany* **2018**, *121*, 833–848, doi:10.1093/aob/mcx141.
23. Yan, H.; KANG, M.Z.; DE REFFYE, P.; DINGKUHN, M. A Dynamic, Architectural Plant Model Simulating Resource-dependent Growth. *Ann Bot* **2004**, *93*, 591–602, doi:10.1093/aob/mch078.
24. Jallas, E.; Sequeira, R.; Martin, P.; Turner, S.; Papajorgji, P. Mechanistic Virtual Modeling: Coupling a Plant Simulation Model with a Three-Dimensional Plant Architecture Component. *Environ Model Assess* **2009**, *14*, 29–45, doi:10.1007/s10666-008-9164-4.

25. Albasha, R.; Fournier, C.; Pradal, C.; Chelle, M.; Prieto, J.A.; Louarn, G.; Simonneau, T.; Lebon, E. HydroShoot: A Functional-Structural Plant Model for Simulating Hydraulic Structure, Gas and Energy Exchange Dynamics of Complex Plant Canopies under Water Deficit—Application to Grapevine (Vitis Vinifera). *in silico Plants* **2019**, *1*, diz007, doi:10.1093/insilicoplants/diz007.
26. Kahlen, K.; Wiechers, D.; Stützel, H. Modelling Leaf Phototropism in a Cucumber Canopy. *Functional Plant Biol.* **2008**, *35*, 876–884, doi:10.1071/FP08034.
27. Thornby, D.; Spencer, D.; Hanan, J.; Sher, A. L-DONAX, a Growth Model of the Invasive Weed Species, *Arundo Donax* L. *Aquatic Botany* **2007**, *87*, 275–284, doi:10.1016/j.aquabot.2007.06.012.
28. Chen, T.-W.; Nguyen, T.M.N.; Kahlen, K.; Stützel, H. Quantification of the Effects of Architectural Traits on Dry Mass Production and Light Interception of Tomato Canopy under Different Temperature Regimes Using a Dynamic Functional–Structural Plant Model. *Journal of Experimental Botany* **2014**, *65*, 6399–6410, doi:10.1093/jxb/eru356.
29. Poirier-Pocovi, M.; Lothier, J.; Buck-Sorlin, G. Modelling Temporal Variation of Parameters Used in Two Photosynthesis Models: Influence of Fruit Load and Girdling on Leaf Photosynthesis in Fruit-Bearing Branches of Apple. *Annals of Botany* **2018**, *121*, 821–832, doi:10.1093/aob/mcx139.
30. Chen, T.-W.; Cabrera-Bosquet, L.; Alvarez Prado, S.; Perez, R.; Artzet, S.; Pradal, C.; Coupel-Ledru, A.; Fournier, C.; Tardieu, F. Genetic and Environmental Dissection of Biomass Accumulation in Multi-Genotype Maize Canopies. *Journal of Experimental Botany* **2019**, *70*, 2523–2534, doi:10.1093/jxb/ery309.
31. Xu, L.; Henke, M.; Zhu, J.; Kurth, W.; Buck-Sorlin, G. A Functional–Structural Model of Rice Linking Quantitative Genetic Information with Morphological Development and Physiological Processes. *Annals of Botany* **2011**, *107*, 817–828, doi:10.1093/aob/mcq264.
32. Groer, C.; Kniemeyer, O.; Hemmerling, R.; Kurth, W.; Becker, H.; Buck-Sorlin, G. A Dynamic 3D Model of Rape (Brassica Napus L.) Computing Yield Components under Variable Nitrogen Fertilization Regimes. **2007**.
33. Boudon, F.; Persello, S.; Jestin, A.; Briand, A.-S.; Grechi, I.; Fernique, P.; Guédon, Y.; Léchaudel, M.; Lauri, P.-É.; Normand, F. V-Mango: A Functional–Structural Model of Mango Tree Growth, Development and Fruit Production. *Annals of Botany* **2020**, *126*, 745–763, doi:10.1093/aob/mcaa089.
34. Kang, F.; Letort, V.; Magaldi, H.; Cournede, P.-H.; Lecoeur, J. SUNLAB: A Functional-Structural Model for Genotypic and Phenotypic Characterization of the Sunflower Crop. In Proceedings of the 2012 IEEE 4th International Symposium on Plant Growth Modeling, Simulation, Visualization and Applications; October 2012; pp. 192–199.
35. Buck-Sorlin, G.; de Visser, P.H.B.; Henke, M.; Sarlikioti, V.; van der Heijden, G.W.A.M.; Marcelis, L.F.M.; Vos, J. Towards a Functional–Structural Plant Model of Cut-Rose: Simulation of Light Environment, Light Absorption, Photosynthesis and Interference with the Plant Structure. *Annals of Botany* **2011**, *108*, 1121–1134, doi:10.1093/aob/mcr190.
36. Pagès, L.; Bécel, C.; Boukcim, H.; Moreau, D.; Nguyen, C.; Voisin, A.-S. Calibration and Evaluation of ArchiSimple, a Simple Model of Root System Architecture. *Ecological Modelling* **2014**, *290*, 76–84, doi:10.1016/j.ecolmodel.2013.11.014.
37. Schnepf, A.; Leitner, D.; Landl, M.; Lobet, G.; Mai, T.H.; Morandage, S.; Sheng, C.; Zörner, M.; Vanderborght, J.; Vereecken, H. CRootBox: A Structural–Functional Modelling Framework for Root Systems. *Annals of Botany* **2018**, *121*, 1033–1053, doi:10.1093/aob/mcx221.
38. Barczi, J.-F.; Rey, H.; Griffon, S.; Jourdan, C. DigR: A Generic Model and Its Open Source Simulation Software to Mimic Three-Dimensional Root-System Architecture Diversity. *Annals of Botany* **2018**, *121*, 1089–1104, doi:10.1093/aob/mcy018.

39. Postma, J.A.; Kuppe, C.; Owen, M.R.; Mellor, N.; Griffiths, M.; Bennett, M.J.; Lynch, J.P.; Watt, M. OpenSimRoot: Widening the Scope and Application of Root Architectural Models. *New Phytologist* **2017**, *215*, 1274–1286, doi:https://doi.org/10.1111/nph.14641.
40. Javaux, M.; Schröder, T.; Vanderborght, J.; Vereecken, H. Use of a Three-Dimensional Detailed Modeling Approach for Predicting Root Water Uptake. *Vadose Zone Journal* **2008**, *7*, 1079–1088, doi:10.2136/vzj2007.0115.
41. Diggle, A.J. ROOTMAP—a Model in Three-Dimensional Coordinates of the Growth and Structure of Fibrous Root Systems. *Plant Soil* **1988**, *105*, 169–178, doi:10.1007/BF02376780.
42. Zhang, W.; Wang, G.; Han, J.; Li, F.; Zhang, Q.; Doonan, J. A Functional-Structural Model for Alfalfa That Accurately Integrates Shoot and Root Growth and Development. In Proceedings of the 2018 6th International Symposium on Plant Growth Modeling, Simulation, Visualization and Applications (PMA); November 2018; pp. 134–140.
43. Barillot, R.; Chambon, C.; Andrieu, B. CN-Wheat, a Functional–Structural Model of Carbon and Nitrogen Metabolism in Wheat Culms after Anthesis. I. Model Description. *Annals of Botany* **2016**, *118*, 997–1013, doi:10.1093/aob/mcw143.
44. Zhou, X.-R.; Schnepf, A.; Vanderborght, J.; Leitner, D.; Lacointe, A.; Vereecken, H.; Lobet, G. CPlantBox, a Whole-Plant Modelling Framework for the Simulation of Water- and Carbon-Related Processes. *in silico Plants* **2020**, *2*, diaa001, doi:10.1093/insilicoplants/diaa001.
45. DeJong, T.M.; Da Silva, D.; Negron, C.; Cieslak, M.; Prusinkiewicz, P. The L-ALMOND Model: A Functional-Structural Virtual Tree Model of Almond Tree Architectural Growth, Carbohydrate Dynamics over Multiple Years. *Acta Hortic.* **2017**, 43–50, doi:10.17660/ActaHortic.2017.1160.7.
46. Louarn, G.; Faverjon, L. A Generic Individual-Based Model to Simulate Morphogenesis, C–N Acquisition and Population Dynamics in Contrasting Forage Legumes. *Annals of Botany* **2018**, *121*, 875–896, doi:10.1093/aob/mcx154.
47. Lopez, G.; Favreau, R.R.; Smith, C.; Costes, E.; Prusinkiewicz, P.; DeJong, T.M. Integrating Simulation of Architectural Development and Source–Sink Behaviour of Peach Trees by Incorporating Markov Chains and Physiological Organ Function Submodels into L-PEACH. *Functional Plant Biol.* **2008**, *35*, 761–771, doi:10.1071/FP08039.
48. Schaphoff, S.; von Bloh, W.; Rammig, A.; Thonicke, K.; Biemans, H.; Forkel, M.; Gerten, D.; Heinke, J.; Jägermeyr, J.; Knauer, J.; et al. LPJmL4 – a Dynamic Global Vegetation Model with Managed Land – Part 1: Model Description. *Geoscientific Model Development* **2018**, *11*, 1343–1375, doi:10.5194/gmd-11-1343-2018.
49. Moreno-Cadena, L.P.; Hoogenboom, G.; Fisher, M.J.; Ramirez-Villegas, J.; Prager, S.D.; Becerra Lopez-Lavalle, L.A.; Pypers, P.; Mejia de Tafur, M.S.; Wallach, D.; Muñoz-Carpena, R.; et al. Importance of Genetic Parameters and Uncertainty of MANIHOT, a New Mechanistic Cassava Simulation Model. *European Journal of Agronomy* **2020**, *115*, 126031, doi:10.1016/j.eja.2020.126031.
50. Bertheloot, J.; Cournède, P.-H.; Andrieu, B. NEMA, a Functional–Structural Model of Nitrogen Economy within Wheat Culms after Flowering. I. Model Description. *Annals of Botany* **2011**, *108*, 1085–1096, doi:10.1093/aob/mcr119.
51. Lacointe, A.; Minchin, P.E.H. A Mechanistic Model to Predict Distribution of Carbon Among Multiple Sinks. In *Phloem: Methods and Protocols*; Liesche, J., Ed.; Springer: New York, NY, 2019; pp. 371–386 ISBN 978-1-4939-9562-2.
52. Lobet, G.; Pagès, L.; Draye, X. A Modeling Approach to Determine the Importance of Dynamic Regulation of Plant Hydraulic Conductivities on the Water Uptake Dynamics in the Soil-Plant-Atmosphere System. *Ecological Modelling* **2014**, *290*, 65–75, doi:10.1016/j.ecolmodel.2013.11.025.

53. Wu, L.; McGechan, M.B.; McRoberts, N.; Baddeley, J.A.; Watson, C.A. SPACSYS: Integration of a 3D Root Architecture Component to Carbon, Nitrogen and Water Cycling—Model Description. *Ecological Modelling* **2007**, *200*, 343–359, doi:10.1016/j.ecolmodel.2006.08.010.
54. Jones Jr., J.B. *Plant Nutrition and Soil Fertility Manual*; 2nd ed.; CRC Press: Boca Raton, 2012; ISBN 978-0-429-13081-6.
